# Living in a World With God: An Interpretative Phenomenological Exploration of the Religious Experiences of Five Baptists in Britain

**DOI:** 10.5964/ejop.3119

**Published:** 2022-05-31

**Authors:** James Murphy, Fergal W. Jones, Dennis Nigbur, Kate Gee

**Affiliations:** 1 Canterbury Christ Church University, Canterbury, United Kingdom; Open University Milton Keynes, United Kingdom

**Keywords:** Christianity, interpretative phenomenological analysis, meaning systems, religion, spirituality, worldviews

## Abstract

Religious and spiritual experiences often form significant elements of people’s narratives about their faith and lives, but the impact of these experiences is often neglected in academic studies. This study investigated the connections between perceived experiences of God and beliefs in the lives of five members of a Baptist church in Britain, using data from semi-structured interviews. Interpretative phenomenological analysis (IPA) was used to explore the data and develop 11 recurrent sub-themes, organized into two super-ordinate themes: “Knowing God” and “Living in the World.” There were idiosyncratic differences between the experiences of the participants, but they all perceived God communicating with them and attributed certain events to God’s influence. These experiences developed real and meaningful relationships with God, and the participants’ faith affected every aspect of their lives, shaping their actions, beliefs and daily lived experiences. The participants’ diverse experiences and beliefs created mutually supporting meaning systems (or worldviews) that were much stronger than the individual elements that contributed to them. God was an intrinsic part of the participants’ social reality, and their lived experiences cannot be adequately understood without appreciating the influence of this central aspect of their lives. These findings show the importance of taking a holistic and idiographic perspective when studying religiosity and spirituality. The study also demonstrates IPA is a useful and effective tool for studying lived experiences of religiosity and spirituality and supports its broader use to investigate such phenomena.

Understanding why people believe the things they do, and how their beliefs shape their actions and experiences, is a central concern for psychologists and other social scientists. The relationship between beliefs and experiences can be complex and, to fully understand either, both must be considered. Individuals develop internal understandings of reality from their experiences and these global meaning systems, or worldviews, allow them to function in the world and help them understand subsequent experiences (Bruner, 1990; Murphy, 2017; Park, Edmondson, & Hale-Smith, 2013). Approximately 85% of the global population report having some form of religious belief, and many find religious meaning systems (or worldviews) provide effective answers to existential questions (Park et al., 2013). However, the way these worldviews are acquired and develop is still only partially understood (Barrett & Lanman, 2008; Park, 2013). Understanding how religious traditions transmit, sustain, and transform their beliefs and practices is important not only for understanding religious traditions but can also help develop broader insights into how people’s beliefs develop through their personal and social experiences.

When studying how people experience and understand the world, it is important to recognize the complexity of people’s lived experiences. Lived religion (McGuire, 2008) is diverse and, even within a single community, different individuals can have contrasting understandings and expressions of their shared beliefs. It is not uncommon for individuals to have beliefs that are incongruent with each other (Chaves, 2010) or “theologically incorrect” according to the traditions with which they identify (Slone, 2004). Religiosity and spirituality are also far more than just beliefs: relationships, identity, and practices are crucial elements of them (Day, 2011; Hood, Hill, & Spilka, 2009; McGuire, 2008). However, distinctions between religious, spiritual, and secular beliefs and groups are primarily emic and reflect political or social factors rather than universal conceptual distinctions (Ammerman, 2014; Fitzgerald, 2000; Murphy, 2017). This means that, rather than focusing on contested categorizations, it is more fruitful to examine why individuals and communities find certain beliefs and practices meaningful and/or useful.

The many connections between beliefs, practices, and experiences mean that to understand any of them adequately they must all be considered. A wide range of complementary methodological approaches is also needed to develop a comprehensive understanding of them (Emmons & Paloutzian, 2003; Paloutzian & Park, 2013). Qualitative methodologies can explore important nuances that more nomothetic approaches obscure, investigating the complexity and diversity of lived experiences which cannot be adequately captured using quantitative measures (Davis et al., 2016; Murphy, 2017). This can help develop better theories and advance the psychological study of religion, spirituality, and worldviews (Hood et al., 2009; Johnson, Hill, & Cohen, 2011; Paloutzian & Park, 2013).

We chose to use interpretative phenomenological analysis (IPA; Smith, Flowers, & Larkin, 2009) for this study because it enabled us to explore both how individuals experienced phenomena they considered significant to their beliefs and how they made sense of those experiences. IPA is adept at investigating topics which are “complex, ambiguous and emotionally laden” (Smith & Osborn, 2015, p. 41), and religion is all of these. IPA’s idiographic approach allowed us to investigate both the convergences and divergences between the experiences of each participant, developing a deeper understanding of how multiple events shaped each other and the participants’ interpretations of them.

Numerous previous studies have used IPA to investigate aspects of spirituality and religiosity. These include studies of how religious individuals make sense of certain experiences (e.g., Moss & Snodgrass, 2017; Ward, 2011) and of specific phenomena that are considered spiritual or religious (e.g., Lewis, Sanderson, Gupta, & Klein, 2018; Wilde & Murray, 2010). However, we believe this is the first study to use IPA to explore lived religiosity/spirituality more broadly and from a “meaning systems” perspective (Paloutzian & Park, 2013), examining how the many elements in religious individuals’ lives interact and mutually interpret each other.

This study investigated the experiences of five members of a Baptist church in Britain. It examined how the participants experienced their faith and made sense of their experiences. This approach follows recent calls to focus on meaning making when studying religiosity (e.g., Paloutzian & Park, 2013) and adopts a holistic approach to doing so. The relative lack of previous psychological studies of mainstream Christian experiences within the United Kingdom makes this a valuable case study that can also provide broader insights into how people make sense of their worlds. This study tests, and ultimately demonstrates, the value of idiographic approaches in the psychological study of religion and we hope it encourages others to conduct similar studies in different populations.

## Method

IPA is an idiographic approach that can explore the rich, idiosyncratic detail of individuals’ experiences and how they make sense of them (Smith et al., 2009). It takes the experiences of individuals as its data but acknowledges that subjective experiences are always interpretations and that the emic interpretations of participants are not the only way to understand the phenomena being studied (Pietkiewicz & Smith, 2014).

### Participants

A single church was chosen for the study, to provide a suitably homogenous group (Smith et al., 2009). This church represents a “mainstream” and typical example of Christianity in contemporary Britain. It is situated in a large town in the South East of England, has an ethnically and economically diverse congregation, and is affiliated with the Baptist Union. Five participants were purposively recruited from its congregation via a gatekeeper. Participants needed to be active members of the congregation between 18 and 65 years old, who had been Christians for at least 2 years. Relatively small sample sizes, in comparison to other qualitative approaches, are an important and distinctive element of IPA studies. IPA explicitly rejects the concept of data saturation, and instead idiographically explores the experiences of a purposively chosen sample. Using five participants allowed for a good balance between detailed analysis of each individual case and exploration of idiographic differences between cases (Smith, 2011; Smith et al., 2009).

Sampling in IPA studies draws from relatively homogenous groups but purposively selects participants from within a group to provide diverse perspectives on the phenomena being studied (Smith et al., 2009). This involves an unavoidable trade-off between homogeneity and diversity of experiences when recruiting participants. We believe using a single congregation as the focus for analysis provided sufficient homogeneity, and not excluding any members of the congregation, based on additional demographic restrictions, provided a richer understanding of the community.

The participants (three men, two women) reflected the wide range of backgrounds within the church’s membership and had diverse economic circumstances, ranging from employment as a medical doctor to being an unemployed single mother. Three participants had attended university. All the participants were British, with two from Black, Asian and Minority Ethnic (BAME) backgrounds. Their ages ranged from their 20s to their 50s. Pseudonyms preserve the anonymity of the participants: Gareth, Havir, Ian, Jenny and Karen.

### Data Collection

The first author conducted semi-structured interviews with each participant individually, in private locations agreed with each participant. The interview schedule (see Appendix in Supplementary Materials) covered seven broad areas, each introduced with an open question and supplemented by additional prompts. Interviews began by asking the participant to speak about who they are and their background. They were then asked about their perspective on life and the world, with prompts eliciting the sources of meaning, purpose and value in their lives. Next, participants were asked to tell the interviewer about God and their religious or spiritual activities. Participants were then asked to describe specific experiences of God in detail. The interview ended by exploring whether the participant thought their beliefs had changed during their lifetime and by giving them an opportunity to discuss anything they had not yet mentioned but felt was relevant or important. The interviews ranged from 44 to 93 minutes in length, with a mean of 57 minutes. Participants were briefed prior to their interviews and debriefed afterwards.

### Analytical Process

Audio recordings of each interview were transcribed and imported into NVivo 11, before being analyzed by the first author, in consultation with the other authors. Each transcript was annotated with initial comments and reflections before coding. Coding was inductive and iterative, first examining each case individually and then re-examining them in light of each other (Smith et al., 2009). Emergent themes were consolidated, and those found in at least half the accounts were identified as recurrent sub-themes and grouped conceptually into super-ordinate themes (Smith, 2011). This process was extensive and exhaustive, with the themes repeatedly refined as they were rigorously checked against the data and discussed by the research team. Writing up qualitative research is an integral part of the analysis (Smith et al., 2009), and our understanding of each theme evolved during this period as we reflexively tested our interpretations of the data. Memos were used throughout data collection and analysis to document this process. The account presented here is an interpretative account of the participants’ experiences and meaning-making processes, based upon the complete data. The extracts presented within it were chosen to illustrate the themes and show key aspects of convergence and divergence within the participants’ experiences.

### Researcher Backgrounds

The research team consisted of the first author, who conducted the interviews and was the principal analyst, and a diverse supervisory team. The first author was raised as an evangelical Christian and has degrees in Theology and the Psychology of Religion, but now identifies as not religious. The supervisory team included a practicing Lutheran, a Humanist Celebrant, and a Mindfulness practitioner. We were committed to engaging with the participants’ experiences in their own terms, putting aside assumptions (both from our own experiences and the extant literature) as much as possible (Smith et al., 2009). We were constantly alert to the possible influence of our own experiences and beliefs on the analysis, and the diversity of the team was particularly useful in this regard. It also helped deepen the analysis and clarify the findings, as the mixture of insider and outsider perspectives ensured many different possibilities were considered. We are confident the findings are strongly grounded in the data because possible interpretations were always rigorously tested against it and many alternative ways of framing the data were considered before reaching the final analysis presented here.

### Ethics

Ethical approval for the study was granted by the university’s ethics committee, and informed consent was received from both the church leadership and each participant. We clearly explained to participants that they did not need to discuss anything that made them uncomfortable; however, all the participants chose to share highly personal accounts of their lives. To protect the participants’ anonymity, the account presented here describes only minimal personal information about them, but the wider contexts of each participant’s life were considered in the analysis. Debriefing provided the opportunity to address any distress or concerns that might have arisen during the interviews, but the participants primarily showed curiosity about the research and expressed gratitude for the opportunity to take part.

## Results

Eleven recurrent sub-themes, each found within the accounts of at least half the participants, were developed and organized into two superordinate themes: “Knowing God” and “Living in the World” (see Table 1). “Knowing God” explores the intimate relationships the participants experienced with God (knowing as relationship with), the ways they made sense of their experiences (knowing as knowledge about), and the certainty of their convictions (knowing as certainty). “Living in the World” explores the tensions of living life “in but not of” a world that is not Christian. It examines the multifaceted ways their faith affected, and was affected by, a range of social and cultural influences. Each sub-theme occurred in the accounts of all five participants, except “Studying scripture to understand God” which was not found in Jenny’s interview. These themes were complementary, and sub-themes often had connections to many others.

**Table 1:** Living in a World With God: Summary of Themes with Example Quotes

Theme: Knowing God	
Sub-theme	Example quote
Experiencing a loving and nurturing relationship with God	“Jesus is my homeboy.” (Karen)
Studying scripture to understand God	“The Bible would be the main way I think I know God, so, as He is revealed in His Word.” (Ian)
Talking to God as a daily companion	“If I see something, I pray about it. And if I need to do something, just to pray that God will help me…” (Jenny)
Connecting to God through worship	“When you worship, God comes to you… you have such a clear connection with God…” (Karen)
Feeling God’s touch and guidance	“[God is] like a light. He’s a light and you feel warm in yourself…” (Jenny)
Seeing God’s hand in events	“[What] has sustained that belief over the years has been a remarkable coming together of events… some would call them coincidences. I would call them answers to prayer.” (Gareth)
Theme: Living in the World	
Sub-theme	Example quote
Being supported by other Christians	“He made being a Christian very fun for a child… he had a lot of influence on me, um, on my Christian beliefs…” (Karen)
Interactions with other perspectives influence beliefs and behaviors	“I started really kind of searching, comparing, you know, my beliefs, my faith, with that of someone [from a] … polytheistic background and faith.” (Havir)
Responding to challenging situations	“Some of them were struggling in their failings and their fallen-ness and therefore my preaching became very much about trying to affirm people in who they are.” (Gareth)
Finding meaning and purpose	“There is something more than what’s in it for me, in the sense of the bigger picture, which certainly adds purpose.” (Ian)
Faith affects everything	“God’s the center… everything flows down from that.” (Havir)

### Theme: Knowing God

All five participants described experiencing a loving and nurturing relationship with God. They considered their relationships with God, and the fact that they knew God personally, a central and defining feature of their Christian identities and the most important thing in their lives. Being Christian, rather than being Baptist, was the cornerstone of their personal and communal identities. There were many similarities between the relationships the participants had with God, but also some individual differences in how they experienced and made sense of their lives and faith. Ian described how, “There’s a God who I know and have a personal relationship with… He’s probably more ‘Daddy’ rather than ‘God’.” Karen’s relationship with God shared the intimacy of Ian’s and she declared joyously that, “Jesus is my home boy.” He was a friend who was always there for her, somebody who she thought was “a lot like us, but, um, just more. He’s just more.” The participants described knowing God in their lives through a range of experiences, and both prayer and worship made them feel close to God.

The participants prayed in a variety of different ways, including formal intercessions during religious services and more informal prayers at other times. Jenny summed up her attitude to prayer by saying: “If I see something, I pray about it. And if I need to do something… I just… pray… anywhere.” This approach was common to all five participants, and shows the importance they placed upon God’s guidance, their belief that God acts in the world, and the intimacy of their relationship with God. The participants believed God was interested in every aspect of their lives, and they could approach him at any time and without formality. They experienced God as a daily companion whom they could always talk to. Despite this intimacy, their “conversations” with God were usually one-sided, and only Ian reported an experience of God speaking to him audibly.

The participants frequently requested guidance or divine intervention in situations, but they reported the outcomes of such intercessions were mixed. Their memories of positive outcomes appeared most salient, and these seemed to mitigate the potential effects of negative outcomes on their faith. Gareth summarized this attitude, saying: “although there are many times when prayers don’t get answered as you would wish, and things don’t go according to plan, the fact that there are enough times when things have worked out… [means you can] make sense of the disappointments by recognizing that there have been many times when those things have happened.”

Musical worship (singing devotional songs) was also an important part of the participants’ lives, and they described how it created a more intimate and emotional connection between them and God than prayer. Like prayer, this worship was not limited to church services. The participants regularly played Christian music in their cars, homes and other contexts. Karen described how “during worship, you’re communicating directly with God… when you pray, you come to God, but when you worship, God comes to you… it does feel like you have such a clear connection with God when you are really enjoying worship.” The participants found joy in their relationship with God, which encouraged them to invest in it, and the experience of clarity made them confident their faith was true.

The Bible played an important role in the lives of all five participants. They considered it to be God’s revelation to mankind, and they studied scripture to understand what God was like and what God wanted from them. Ian summarized this position, saying: “the Bible would be the main way I think I know God… he is revealed in His Word.” Despite the importance of their personal experiences, the Bible had central importance in their lives because it helped them make sense of their experiences. As Karen said, “everything that we need to know, how to live, to become closer to Him, I think it’s all in the Bible.” Only Jenny, who was dyslexic and struggled to read and study, did not speak about reading the Bible for herself—but she still valued its contents and learned about it through sermons and conversations with other Christians. For the participants, the Bible was both a source of information about God and a way God communicated with them.

The participants described often having tangible feelings of God’s presence and attributed certain thoughts to his inspiration and guidance. These experiences played a crucial role in affirming and reinforcing their beliefs about God and their relationship with him. The participants’ feelings of God’s presence were somewhat ineffable, but tangible, somatic and emotional. Ian described them as: “a really, really ecstatic, but in control, happy feeling—almost like the nice warm glow you get if you’ve had one drink too many… It’s warm, fuzzy… hard to describe.” These feelings of joy, affection and gratitude were experienced by all the participants in a wide range of circumstances and they could both affirm and stimulate faith. Gareth described how his conversion at a Billy Graham rally involved such a bodily response. After the sermon, during the altar call, Gareth’s “head changed. [I felt] … tingles, all the rest of it… and that was what converted me.” Believing he had experienced God’s touch provided a powerful affirmation that God was real, active and cared about him.

The participants also spoke about “just knowing” that some of their thoughts were from God. These inspired thoughts could take many forms, including recalling Bible verses, but they all involved a conviction they were (or at least might be) from God and should be heeded. Ian described this feeling as “a sense of certainty, a sense of intuition, or maybe word of knowledge might be a way to describe it.” Despite the conviction they often felt, the participants were frequently hesitant to ascribe these thoughts to divine inspiration and did not do so uncritically. They sought the input of trusted others and compared their thoughts to scripture for confirmation. Jenny described this process, saying, “I don’t feel I hear God that much, but I do, because people say to me, yeah, that’s from God… I need a second opinion before, before I realize it’s God.” Gareth also acknowledged the potential to be wrong about what God was saying in a situation, attributing such errors to the need for God to speak “through the filters and the impressions of our minds and because of that we sometimes will get it wrong.” This lack of certainty and clarity about particular instances of God speaking helps explain the iterative way that their relationships with God, and beliefs, developed. They all believed they experienced God’s touch and guidance, but this had an inherent ambiguity that required them to integrate many different experiences into a more coherent set of beliefs.

The participants also described how they saw God’s activity in the events of their lives. These experiences nurtured and reinforced their beliefs about God, particularly the beliefs that he was real and cared about them. Gareth described how, “One of the things that has sustained that belief over the years has been a remarkable coming together of events. Some would call them coincidences. I would call them answers to prayer.” All five participants told stories about unlikely but meaningful circumstances that helped shape their lives. These ambiguous events included people finding jobs after long searches and medical issues being less severe than they might have been. For the participants, these experiences demonstrated that God answered their prayers and kept his promises, helping them have faith that they knew God’s character and that they were his people. Karen highlighted the importance of these experiences of divine intervention for the participants, reporting she regularly told people the reason she knew God was real because she had “experienced God in my life even before I was a Christian.”

Many of the events that the participants described were relatively mundane, but they also included the apparently miraculous. Ian reported the most dramatic of these experiences, saying that, “God’s protected me. Supernaturally, a couple of times… there was a sense, you know quite miraculously—God’s protecting us.” The story he told to illustrate this involved an angry confrontation with a soldier wielding a grenade and the unexpected intervention of a bystander who defused the situation. For Ian, this was a clear example of divine intervention, although a more mundane interpretation is also possible and the extent to which participants were comfortable acknowledging the ambiguity of the situations varied. These experiences, and retelling them to others, created a sense that the participants lived lives in which God was an active part.

The participants’ relationships with God, although loving and nurturing, also included negative emotions and disagreements. The participants described experiencing God asking them to do things they did not want to do as well as times when God did not do what they wanted. For example, Karen discussed how she could be “a little hardened, or stubborn… He might be giving me little signals, do this, do this, and I’m like, I don’t want to do that!” These arguments and disagreements with God did not appear to diminish the bond that the participants experience with him, but instead seemed to ultimately strengthen their faith in God when they experienced positive outcomes. For example, when a serious relationship Karen was in ended, she was angry and thought God had let her down, but “a couple of years later, I met my husband. And I was, like, oh, I get it now… I’m sorry, Lord. You were working it out for me and I got mad at you. I’m really sorry.” The language used to describe these experiences demonstrated both the intimacy and vibrancy of the relationships. The participants believed they knew God intimately and with certainty; they related to him like a close friend whom they trusted. The participants’ diverse experiences of God in their lives affirmed their faith and provided a bulwark against doubts and tribulations.

### Theme: Living in the World

The personal and dynamic relationships with God that each participant reported experiencing did not occur in isolation. The participants described living lives that were varied and social, involving active engagement with all aspects of modern life. The participants’ interactions with other people, and involvement in a wide range of situations, influenced how they experienced God and life in various ways.

Other Christians appeared to play a crucial role in supporting and developing the participants’ faith and relationships with God. The participants discussed being members of multiple churches at different stages of their lives, and these communities influenced what they believed and did. Close friendships and romantic relationships with other Christians were particularly important to the participants, providing support and affirming their faith in their daily lives. God had a central role within their family lives and teaching their children about their faith encouraged them to live out their own beliefs. Havir summed this up, reflecting that, “it is so important to steer them in the right direction… the way Jesus wants us to lead our lives. I think those truths have been drummed home a lot more because we want the kids to be like that.” Living with other Christians helped the participants keep God at the center of their lives and develop into the people that they wanted to become. They viewed these influences as positive blessings, understanding processes as supportive of their efforts to develop into the people that they wanted to become.

Gareth’s descriptions of his evolving faith clearly showed the importance of other Christians in his life. He described how, before he became a Christian, persistent friends at university, “took me to church on Sunday nights… I’d never experienced anything like that… To encounter this whole generational thing, that young people could believe this and live this was, was significant.” The passion and commitment of his friends showed Gareth a type of Christianity he had never encountered before, offering an alternative way of understanding the world that intrigued him. His experiences of them led him to attend the service that changed his life and they helped him make sense of his experiences afterwards. After he graduated, Gareth was surrounded by Christians with different beliefs, and he attributed his own beliefs becoming more inclusive and liberal to their influence. Reflecting on his experiences, he said, “You’re not conscious at times of the subtle changes in your thinking that are taking place… I can look back and think of some… wonderful prayers and preaching… [that] contribute[d] to a re-formation of the theological mindset and approach.” Both the explicit teaching and the implicit values of the participants’ communities appeared to influence how they perceived themselves, God, and the wider world.

The participants’ closest family and friends shared their beliefs, but they lived in a society that increasingly did not. Their experiences and beliefs were clearly also influenced by the wider society around them in various ways. Encounters with behaviors and beliefs that conflicted with their own could lead to the participants altering their beliefs or becoming more convinced of them.

Havir, who went through an extended period of more nominal faith despite being raised in a Christian family, described how a relationship with a Hindu girlfriend caused him to decide what he believed and what he wanted from life. He “started comparing… really kind of searching, comparing, you know, my beliefs, my faith, with that of someone… [with a] polytheistic background and faith.” His girlfriend presented him with the possibility of an alternative way of life and made Havir need to choose how he wanted to raise his future family. This reflection was the catalyst for a profound change in the importance he placed on his religion and how he lived his life.

The participants’ encounters with other perspectives sometimes caused them to change some of their beliefs. For example, Ian described how his beliefs about homosexuality had shifted over the previous decade and he identified friendships with gay men as a key catalyst for that change. He suggested that the liberal attitudes of wider British society may have influenced his beliefs on the issue, and this caused tension for him. He reflected that, “there is a worry… about just, you know, the church following society's line.” For the participants, God and the Bible were supposed to determine what was right and they were supposed to be different even when it carried a cost. However, their interactions with others sometimes caused them to question aspects of their faith, and this process could lead to them either altering their beliefs or becoming more convinced of them.

Experiencing challenging situations and events often drew the participants closer to God, who provided security and comfort in times of crisis. Jenny had experienced many traumatic experiences, including severe physical illness, poverty, and abuse. She described how God was “a light… and he helps you in difficult times.” She also viewed her suffering as having purpose and value because it helped her get “to the point where I had to rely on God. Um, I didn’t do that before. It took two years in this horrible place. I had to learn stuff.” Like the other participants, she looked for positives in her experiences of adversity and gave God credit for the situations not being even worse. The process was neither quick nor easy, but she learned to turn to God in her suffering and the comfort she found strengthened her relationship with God.

The participants spent considerable amounts of time helping others, and they described how these relationships made their lives more meaningful. Jenny described how helping others gave meaning to the many struggles she had been through. She now worked with a church-based charity and said she had “come to the conclusion that… If I wasn’t bullied and I didn’t go through what I did, I wouldn’t have the real experience to tell people… [God] has changed… the challenging bits to, OK, positives.” The participants’ faith helped them find meaning and purpose both in their ongoing lives and in their pasts. They believed they were called by God to do what they were doing, and this enabled them to view their daily activities as sacred and significant. The belief that what they were doing truly mattered helped transform mundane activities into something more and nurtured their sense of living lives that were sacred and meaningful.

Challenging encounters or events sometimes posed difficult questions for the participants and required them to reconcile their beliefs with the world they experienced. For example, the participants sometimes found suffering and pain theologically challenging to reconcile with their conception of a loving and omnipotent God. Gareth described how seeing the pastoral needs of others, as they lived through difficult situations, led to him softening his doctrinal beliefs. Compassion led him, “to rethink [his theology], because I need to be able to affirm these people as legitimate, bona fide members of my congregation… they need to be affirmed… therefore, my preaching became very much about trying to affirm people in who they are.” These changes did not stop the participants from being Christian, but they altered what they understood “being Christian” to mean and they viewed these changes as their faith maturing. Challenging circumstances sometimes functioned as catalysts for change in the participants’ lives, leading to significant and lasting changes in their beliefs and behaviors.

The participants believed the calling to live their lives for God was an inherent part of being a Christian and that their actions and choices had eternal significance. Being Christian was a central facet of their personal identities and lives. Ian described how his faith gave him a sense of “the bigger picture, which certainly adds purpose.” Havir went further, saying that he believed that without God, and an eternal afterlife, things would be “futile” and “meaningless.” The participants cared deeply about other people, but their faith altered their perspectives and affected their choices. It framed how they understood themselves and the world.

For these five participants, their relationships with God and Christian faith affected their entire lives. Their faith influenced the activities they did, the choices they made, and the relationships they had. Havir summed this up, saying: “God’s the center. That’s it, really… everything flows down from that.” The boundary between the secular and the sacred was at least blurred, if not meaningless, in the participants’ lives because they included God in every situation and interpreted events through their faith. Ian described how “worship is all our life, and everything that we do and sacrifice to God,” and Gareth noted he could not “separate faith from life, because to me they are entwined.” These participants’ faith influenced everything they did; they experienced God as a supporting presence in every area of their lives. In Ian’s words, their faith was not a crutch but was “more like a mountaineer’s pole—it helps you, but because of it you can climb mountains that you wouldn’t otherwise climb.”

## Discussion

This study used IPA (Smith et al., 2009) to explore the lived experiences of five members of a Baptist church in Britain. The participants described experiencing relationships with God, similar to their relationships with other humans. These relationships between the participants and God were a fundamental part of their lived experiences and affected all aspects of their lives. They believed they knew God intimately and described living in a world of which God was an active and integral part. The participants’ relationships with other people, their life choices, and their overall wellbeing were all connected to, and rooted in, their belief that they knew God as a living presence in their lives. Without understanding the participants’ faith and the relationships they experienced with God, any understanding of the participants’ lives and behavior would be incomplete.

### Living in a Sacred World

The experiences that participants attributed to God were diverse, and there were not clear boundaries between what the participants considered sacred and the rest of their lives. Any experience could encourage them to pray, and they could perceive any situation as the work of God. These findings echo Ammerman’s (2014) work in the United States, that found religion often permeates social boundaries to both influence and be influenced by interactions in other spheres of individuals’ lives. As Taylor (2007) notes, religious beliefs are often not something extra that is “added on” to a secular life. For many people, religious identities and beliefs are an integral part of how they understand themselves and the world, affecting many aspects of their lived experiences. The participants’ faith provided the interpretative framework for their whole lives.

The participants’ experiences of such a sacralized world are unlikely to be shared by all Christians. More nominal Christians, who do not frequently attend church or participate in other religious practices, probably do not have similar relationships with God or the same experience of living in a world where God is active as these participants. Havir’s experiences demonstrate how someone can understand being a Christian in disparate ways at different points in their lives. In contrast to his more recent experiences, for many years his faith had little influence on his life. This fits with the idea that each individual believer constructs what it means to be a Christian out of the many cultural resources they have available, and these personal meaning systems do not always give a prominent place to God or broader existential ideas (Lee, 2015; Murphy, 2017). How people understand themselves and the world changes during their lives, and significant changes in individuals’ worldviews can occur more than once (Fowler, 1981; Streib & Keller, 2004).

The idiosyncratic blending of diverse beliefs and practices by each individual means that care must be taken when using a fractionating approach (White, 2017; Whitehouse & Lanman, 2014) to study specific religious phenomena. The combined effects of the diverse elements of an individual’s meaning system may be greater than the sum of their individual practices and beliefs. Both prayer and worship were important to the participants, but it was the combination of the two practices, with their broader beliefs and social contexts, that sustained the participants’ faith and guided their actions. God was an integrating focus and an intensive concern for participants, with extensive effects throughout their life (Bailey, 1999). It is clearly important to study individual “religious” phenomena, but how these phenomena are experienced, the meaning ascribed to them, and the effects they have may both influence and be influenced by a wide range of other beliefs and related experiences.

### Constructing a Sacred World

The importance of socialization in the formation and development of religious beliefs is well-established (Beit-Hallahmi & Argyle, 1997; Hood et al., 2009), and the influence of social and cultural factors was clear in the accounts of all the participants. Most people accept the existential interpretations and explanations of those they trust (Altemeyer & Hunsberger, 1997; Granqvist & Kirkpatrick, 2004), though as this study shows they do not always do so uncritically, and experiences may cause them to question or reject such explanations. God was a real and important part of the social reality (Berger & Luckmann, 1967) the participants shared, and their faith in him had wide-reaching consequences. Understanding their lives and actions without recognizing the role God played in them is impossible.

The participants, both as individuals and a group, interpreted events in ways consistent with, and reinforcing, their shared worldview (Johnson et al., 2011). Ambiguous events were often attributed to divine intervention (Spilka, Shaver, & Kirkpatrick, 1985), and the encouragement and support of trusted others helped overcome any doubts they experienced (Taves, 2017). Their frequent declarations and affirmations of the truth of their relationships with God helped create their social reality (Searle, 2010). The cumulative effects of many experiences also made each of them more persuasive than they would have been in isolation, repeatedly reinforcing their trust in God. The participants’ world became sacred through their communal practices and expressions of faith (Taves, 2009).

The interactions of different influences, both Christian and non-Christian, contributed to the idiosyncratic beliefs of each participant, which sometimes were incongruent or “theologically incorrect” (Chaves, 2010; Slone, 2004). As Lee (2015) argues, people often draw upon a range of existential resources to help them make sense of their own lives and experiences. The differences between participants’ experiences should not be overlooked, as they are crucial to understanding their beliefs and behaviors. The five participants were part of the same church and espoused the same doctrinal beliefs, but they had very different lived experiences that influenced their physical and psychological lives. The accounts of the participants support viewing beliefs about God, the self and the world as part of a cohesive, idiosyncratic, model of the world that each person develops during their life in response to their experiences (Murphy, 2017).

### Experiencing a Relationship with God

The experience of knowing God directly was of central importance to each participant and provided the bedrock for the participants’ faith and beliefs. The relationships between the participants and God developed over time and, like most relationships, had both negative and positive features. The participants described their relationships with God as being like a relationship with a close friend or family member, and it is plausible that this similarity to other relationships reinforced their belief that it was with a real and independent being.

God, for these participants, was a non-human supernatural agent whom they seemingly perceived and experienced using very similar cognitive mechanisms to other agents in the participants’ worlds (Boyer, 2002). They found his existence not only plausible but actually indisputable, because of the combination of their frequent personal experiences and the supporting experiences of those around them. The findings of this study do not speak to the ultimate truth, or otherwise, of the participants’ interpretations of their experiences, and we wish to note the data are consistent with a range of possibilities. These include both that the participants are genuinely experiencing something beyond themselves and the view that religious experiences are by-products of our evolved cognitive architecture, cultural contexts and ambiguous sensory experiences (Kirkpatrick, 2013).

The participants described God as a source of personal comfort and security. They experienced God as a reliable, powerful, and loving presence in their lives. Their differing childhood experiences and the comfort they derived from their relationships with God are consistent with the theory that God can function as either a surrogate or complementary attachment figure for different religious individuals (Granqvist & Kirkpatrick, 2004; Kirkpatrick & Shaver, 1990). The participants were grateful they experienced God as a supportive presence in their lives, and they shared their faith with others because they wanted them to benefit from the same experiences.

The accounts of how the participants learned to experience God’s presence in their lives are also consistent with the process of metakinesis, as conceptualized by Luhrmann (2004, 2012). This process involves learning to identify sensations as signs of God’s presence and using these attributions to develop relational practices. The participants described learning to hear and feel God better, engaging in regular spiritual activities and being guided by those around them to interpret certain experiences as God’s presence. The participants’ experiences of God and sense-making processes were embodied, active, and situated in their social context (Larkin, Eatough, & Osborn, 2011). The participants’ experiences shaped their beliefs, but these experiences were themselves shaped by their beliefs and by the beliefs of those around them in an active, iterative and embodied process. This process is ontologically ambiguous, and neither assumes nor denies the validity of individuals’ emic interpretations of their experiences.

Fowler (1981) noted that people tend to love and value that which loves and values them. These participants came to know God through experiences of love and mercy. They responded with love and service of their own. The Bible and the traditions of their church helped shape the participants’ knowledge of God, but their personal experiences of God through these things was especially influential. Emotional, intellectual and social factors supported each other and gave the participants a robust and cohesive experience of living with their God. Their experiences of being in a loving relationship with God and feeling his touch in their lives assured the participants of the reality they believed in. The support of other Christians helped develop these beliefs and provided encouragement and guidance in times of crisis. The combination of these many different elements of their lives helped them to grow closer to God and sustain their faith, enhancing their subjective wellbeing and lives.

### Limitations and Future Directions for Research

This study does not claim to describe the experiences of all Baptists in Britain, let alone all Christians. IPA does not attempt to produce overarching descriptions of the experiences of a broad group. Instead, it develops understandings of how individuals experience and make sense of complex phenomena by exploring idiographic details and contextualizing them in ways that refine and illuminate our understanding of phenomena. There were variations between the experiences of different participants, as well as between the experiences of individual participants at different times in their lives, that might have been masked by quantitative or nomothetic approaches. Our analysis of the data suggests these nuances are important for understanding not only the current experiences and beliefs of the participants, but also how and why they have changed during their lives. IPA’s strong idiographic commitment is a strength that enhances the analysis, rather than a weakness.

Future research exploring religiosity, spirituality, and meaning-making should consider the broad range of things that can influence individuals’ beliefs and experiences, examining the different ways these influences and experiences are integrated by individuals. This study demonstrates the importance of the interactions between many different experiences in the development of individuals’ worldviews and shows the role that seemingly mundane experiences can play in shaping and sustaining beliefs. Researchers should also be sensitive to both the diversity of experiences between individuals and the different ways that seemingly similar experiences can be made sense of by different individuals, or by the same individual at different times.

IPA’s reliance on a double hermeneutic, where interpretations of experiences are unavoidably filtered through both the participants and researchers, means it is inherently subjective. This subjectivity is not unique to qualitative research, but it is important to note the broader beliefs and culture of both the participants and researchers shaped the data and its analysis. The diversity of the research team and its specialist expertise helped enhance the quality of the analysis. The first author’s intimate understanding of the language and theological beliefs of the participants provided a strong starting point for the analysis, but this was supported by careful and iterative probing of the participants’ meaning both during the interviews and the subsequent analysis. The strong rapport that developed in the interviews, shown by the highly personal experiences the participants disclosed, also helped generate rich data and insights. The analysis presented here is, we hope, both convincing and rigorous.

We believe that further studies of other religious groups using IPA (and similar methodologies) are the logical next step from this study, to establish the transferability of our findings. A broad range of studies, each exploring a different group and conducted across diverse cultures, could generate valuable insights into how religiosity and spirituality affect people’s lives and worldviews. The conclusions that can be drawn from any single study are limited, but the aggregation of findings from multiple studies can increase the reliability and transferability of research (Larkin, Shaw, & Flowers, 2019).

## Conclusions

This study demonstrates both the importance of religiosity and spirituality in some individuals’ lives and the valuable contribution that IPA can make to their study. Each participant described a unique relationship with their God that was a central and vivid part of their lived experience. The participants’ relationships with God intertwined with every aspect of their lives, and understanding their experiences requires a holistic perspective that does not view their faith as something that is simply “added onto” an otherwise secular life. The participants’ own experiences, and the supporting experiences of those around them, developed comprehensive worldviews that allowed them to make sense of their lives. Gareth, Havir, Ian, Jenny and Karen, not only lived in a world where their God existed, they lived in that world with their God.

## Supplementary Materials

For this article the following Supplementary Materials are available via PsychArchives (for access see Index of Supplementary Materials below).

- Appendix: Annotated Interview Schedule (The Self and The Sacred)

MurphyJ.
JonesF. W.
NigburD.
GeeK.
 (2022). Supplementary materials to "Living in a world with God: An interpretative phenomenological exploration of the religious experiences of five Baptists in Britain"
[Appendix: Annotated Interview Schedule]. PsychOpen. 10.23668/psycharchives.6529
PMC963255136348696

## Supplementary Materials

####index will be inserted#####
